# Thyroid tuberculosis in women: a scarce phenomenon, with predominancy in women!

**DOI:** 10.1590/1806-9282.20240767

**Published:** 2024-11-11

**Authors:** Demet Sengul, Ilker Sengul, Feyza Yildiz Aytekin, Ersin Kuloglu, José Maria Soares

**Affiliations:** 1 Giresun University, Faculty of Medicine, Department of Pathology – Giresun, Turkey.; 2 Giresun University, Faculty of Medicine, Division of Endocrine Surgery – Giresun, Turkey.; 3 Giresun University, Faculty of Medicine, Department of General Surgery – Giresun, Turkey.; 4 Ministry of Health Prof. Dr. A. Ilhan Özdemir State Hospital, Department of Infectious Diseases and Clinical Microbiology – Giresun, Turkey.; 5 Giresun University, Faculty of Medicine, Department of Internal Medicine – Giresun, Turkey.; 6 Universidade de São Paulo, Hospital das Clínicas, Faculty of Medicine, Laboratório de Ginecologia Estrutural e Molecular (LIM-58), Department of Obstetrics and Gynecology, Gynecology Discipline – São Paulo (SP), Brazil.

Dear Editor,

*Latet anguis in herba*. The thyroid gland, *per se*, is an unusual site of extrapulmonary tuberculosis (Tbc), even in endemic areas, which emerges in ∼0.1–0.4% of all Tbc lesions. This phenomenon was first reported by Lebert as part of a study on disseminated tuberculosis in 1862^1,2^. Thyroid tuberculosis (ThyTbc) does not possess pathognomonic clinical manifestations, which leads most cases to be diagnosed through postoperative histopathologic examination. ThyTbc can be evaluated into two subdivisions: primary and secondary. Primary ThyTbc is frequently caused by a direct infection of the gland by *Mycobacterium tuberculosis* (*M. tuberculosis*), while secondary arises after an infection with Tbc in any other parts of the body. This phenomenon, in general, might easily be misdiagnosed clinically due to the lack of apparent clinical manifestations and signs within the laboratory and imaging modalities^3^. Herein, we would like to mention the case of a 47-year-old female who was admitted to an external center due to the presence of several thyroid nodules recognized fortuitously on ultrasound as part of a regular check-up. She had no family history of thyroid disease and a pulmonary or extra-pulmonary Tbc without contact with any known people with Tbc. On her head and neck medical history, a euthyroid hormonal status, a mobile, soft, and painless goiter without any palpable, mobile cervical lymph node, an absence of signs of compression to the upper airway and digestive tracts, and a substernal appearance were reported. An ultrasound of the neck region had been realized, which objectified the presence of multiple thyroid nodules in both lobes, involving 25×13 mm in the left and 18×5 mm in the right lobe of the gland without any central or lateral cervical lymph node involvement. The nodule on the left was hypoechoic and solid, including microcalcifications with irregular borders, while the right one was isoechoic, solid, and possessed regular borders. Afterward, she had a thyroidectomy history without any application of a fine-needle aspiration procedure. Surprisingly, the histopathologic evaluation revealed a granuloma with epithelial cells and caseous necrosis. A Zielh Nelsen stain for acid-fast bacilli, utilizing 20% sulfuric acid, exhibited numbers of acid-fast bacilli, confirming the morphology of mycobacteria. Moreover, she had a positive sputum culture for *M. tuberculosis*. The patient was admitted with amedical finding of ThyTbc inferred from her medical history. *A posteriori*, she had undergone 9 months of medical therapy based on isoniazid, rifampicin, pyrazinamide, and ethambutol. Moreover, her follow-ups were performed every 3 months for the first year and every 6 months after that and were based mainly on the clinical examination. Finally, no sign of a complication and/or recurrence has been detected to date for approximately 19 years. As such, *M. tuberculosis* is an aerobic bacillus responsible for tuberculous infection. Every year, World Tbc Day, the day in 1882 when Professor Robert Koch announced his discovery of this bacillus of a microbial agent, *M. tuberculosis*, is commemorated on March 24 to envision raising public awareness of Tbc for being a preventable and treatable disease. Even though effective treatment for Tbc has been available for over 65 years, today it causes 1.3 million deaths yearly, mostly in low- and middle-income countries. As such, today, drug-resistant Tbc is stated as the leading cause of death in view of antimicrobial-resistant disease in the world, with hundreds of thousands of people affected every year^2,3^. The thyroid, this delicate gland^4^, is affected by *M. tuberculosis*, which is a rare condition. This disease can manifest in various ways, and it does not have specific symptoms that are characteristic of ThyTbc. The primary modality for establishing the diagnosis is fine-needle aspiration with subsequent bacteriologic or biologic investigation. All age groups are affected, with a predominance of women and a mean age between 30 and 46 years^5^. As such, the rarity of this delicate gland and the relative resistance of the gland to *M. tuberculosis* have been expressed in terms of good oxygenation, therefore abundant vascularization, and the bacteriostatic properties of the thyroid hormones. Nevertheless, advanced age, diabetes mellitus, immunosuppression, and malnutrition may contribute to ThyTbc development^4-6^. Mycobacterial infection leads to chronic and persistent inflammatory processes. Mycobacterial cell wall components have been reported to induce DNA damage through the production of nitric oxide and reactive oxygen species, the damage of which has been linked to inflammation and carcinogenesis^7,8^. Chronic inflammatory conditions frequently use multiple mechanisms to generate an appropriate microenvironment for malignant development. As such, mycobacterial infection results in chronic and persistent inflammation. Zhang et al.^9^ stated that it also induced anti-apoptotic activity by upregulating B-cell lymphoma two gene expression. Rangel Moreno et al.^10^ reported elevated levels of prostaglandins following a mycobacterial infection. Consequently, some authors put forward that the combination of direct DNA damage, inhibition of apoptosis, and chronic inflammation might give rise to a highly conducive microenvironment for tumorigenesis^7^. ThyTbc is challenging to diagnose due to a lack of specific signs and symptoms. Therefore, it may present as a solitary nodule or multinodular goiter, even presenting as a chronic neck abscess. However, the presence of another Tbc lesion or even sequelae might help to ease the accurate diagnosis. In the hormonal status of the aforementioned phenomena, thyrotoxicosis with hyperthyroidism might occur after parenchymal destruction, leading to a massive release of thyroid hormones; thereafter, hypothyroidism may arise due to the destruction within the gland. Although cervical lymph node involvement might occur, it is suggestive of a neoplastic disease of the glands^1-3^. Contrarily, our patient, who was admitted to with a history of thyroidectomy (possessing a histopathologic diagnosis of ThyTbc) due to a mobile, non-tender, and painless multinodular goiter without evidence of hypothyroidism, did not possess any cervical lymph node involvement. ThyTbc is very rare disease, and the positive diagnosis relies on the histologic and/or bacteriologic examinations. Imaging technology is not very useful in engraving diagnosis. This infection may first occur primarily in the thyroid gland or may be secondary to Tbc in other parts of the body^11,12^. Of note, the clinical manifestations of this relevant association are still variable. Variable presentation, frequently resembling that of malignancy or a euthyroid nodular goiter, sparks conflicting controversies for providers in thyroidology^13-29^. Treatment modalities are based on a surgical approach and meticulous anti-Tbc therapy. Herewith, thyroidologists and thyroid health providers must be vigilant while managing thyroid nodules, as there is always the possibility of suspicious nodules or lesions akin to an inflammatory or infectious cause, even in ThyTbc, particularly for women. This issue merits further investigation.
